# Aristolactam-Type Alkaloids from *Orophea enterocarpa* and Their Cytotoxicities

**DOI:** 10.3390/ijms13045010

**Published:** 2012-04-20

**Authors:** Sanchai Nayyatip, Pak Thaichana, Mongkol Buayairaksa, Wirote Tuntiwechapikul, Puttinan Meepowpan, Narong Nuntasaen, Wilart Pompimon

**Affiliations:** 1 Laboratory of Natural Products, Center for Innovation in Chemistry, Faculty of Science, Lampang Rajabhat University, Lampang 52100, Thailand; E-Mails: sanchai_n@hotmail.com (S.N.); bymongkol@gmail.com (M.B.); 2 Department of Biochemistry, Faculty of Medicine, Chiang Mai University, Chiang Mai 53000, Thailand; E-Mails: kappa_f4@hotmail.com (P.T.); wirotetunti@yahoo.com (W.T.); 3 Department of Chemistry, Center for Innovation in Chemistry, Faculty of Science, Chiang Mai University, Chiang Mai 53000, Thailand; E-Mail: pmeepowpan@gmail.com; 4 The Forest Herbarium, Department of National Park, Wildlife and Plant Conservation, Ministry of Natural Resources and Environment, Bangkok 10220, Thailand; E-Mail: narong_1960@hotmail.com

**Keywords:** *Orophea enterocarpa*, Anonaceae, aristolactam, human colon adenocarcinoma cells, cytotoxicities

## Abstract

A new aristolactam, named enterocarpam-III (10-amino-2,3,4,6-tetramethoxy phenanthrene-1-carboxylic acid lactam, **1**) together with the known alkaloid stigmalactam (**2**), were isolated from *Orophea enterocarpa*. Their structures were elucidated on the basis of interpretation of their spectroscopic data. Compounds **1** and **2** exhibited significant cytotoxicities against human colon adenocarcinoma (HCT15) cell line with IC_50_ values of 1.68 and 1.32 μM, respectively.

## 1. Introduction

*Orophea enterocarpa* belongs to the Anonaceae family. In Thailand, this plant, called “Kloew Kang”, can be found in scattered locations of the southern and eastern regions [1]. Orophea plants have been used widely as traditional medicine, for example, the root of *O. setosa* was used to cure coughs or fever [2]. Phytochemical studies on several Orophea species have been reported, resulting in the isolation of fatty acid, lignans, flavonoids and alkaloids [3–7].

A preliminary survey for biological activities of the crude methanol-dichloromethane extract of *O. enterocarpa* demonstrated that the extract exhibited cytotoxicities against lymphocylic leukemia (P-388), human carcinoma (KB), human breast adenocarcinoma (MCF7), human lung cancer (Lu-1), rat glioma (ASK), noncancerous human embryonic kidney (Hex 293) and human bladder (T24) cell lines with EC_50_ values of 0.20, 8.56, <4.00, 12.20, 12.48, 8.51 and 18.69 μg/mL, respectively. This paper deals with the isolation and structure elucidation of compounds **1** and **2** as well as their cytotoxicities against human colon adenocarcinoma (HCT15) cell line.

## 2. Results and Discussion

Successive chromatographic separation of the methanol-dichloromethane extract from the leaves and twigs of *O. enterocarpa* yielded two aristolactam-type alkaloids, a new alkaloid named enterocarpam-III (10-amino-2,3,4,6-tetramethoxyphenanthrene-1-carboxylic acid lactam, **1**), together with stigmalactam (**2**) [8]. The structures of **1** and **2** were established by interpretation of their spectroscopic data.

**Figure 1:**
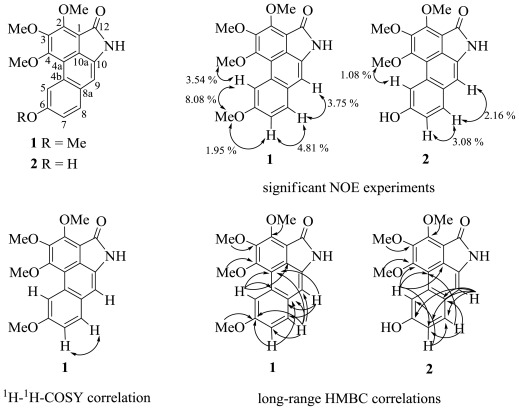
Structures of compounds **1** and **2** together with NOE experiment, significant correlations in the COSY, HMBC spectra.

**Figure 2:**
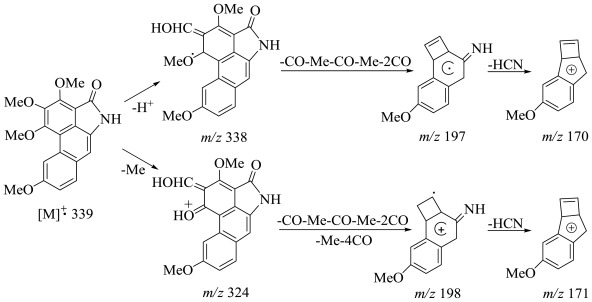
The EIMS mass fragmentations of compound **1**.

Compound **1** had the molecular formula C_19_H_17_NO_5_, deduced from the HRESIMS mass spectrum (found *m/z* 340.1184 [M + H]^+^). The UV spectrum exhibited absorption at λ_max_ nm (log ɛ): 313 (3.17), 359 (2.77), 386 (2.74), 550 (1.74), which corresponded to a phenanthrene chromophore [5]. The IR spectrum showed the presence of an amide group by observation of a pair of fairly strong asymmetric and symmetric N–H stretching absorption bands at 3464 and 3167 cm^−1^, respectively, together with a C–N stretching absorption band at about 1394 cm^−1^. The C=O absorption band partially overlapped the N-H bending absorption band which come into view in the range from 1699 to 1682 cm^−1^, making the C=O absorption band appeared as a doublet. In addition, the aromatic phenanthrene and aralkyl ether moieties were confirmed by the IR bands at (C=C) 1654, 1614, 1568, 1533, 1483, 1475, 1439 and (C–O–C) 1201, 1157, correspondingly. Analysis of the NMR data (Table 1) for **1** immediately suggested a highly aromatized molecule, as the ^13^C NMR chemical shifts suggested that 14 of the 19 carbons were aromatic. The ^1^H-^1^H-COSY and HMBC correlations (Figure 1) identified resonances consistent with a phenanthrene moiety (*δ*_C_ 157.6, 157.1, 154.2, 146.0, 131.8, 129.7, 127.8, 127.7, 126.1, 116.1, 115.9, 109.5, 108.5; *δ*_H_ 8.72, 7.71, 7.18, 7.16) as well as amide carbonyl (*δ*_C_ 167.7) and four methoxy (*δ*_C_ 63.1, 61.7, 60.8, 55.4; *δ*_H_: 4.50, 4.19, 4.01, 3.99). ^1^H NMR signals pattern in the aromatic region showed the presence of two singlets at *δ* 8.72, 7.16 and two *ortho* coupled protons at *δ* 7.18 with *δ* 7.71, respectively. It also showed four 3H singlets at *δ* 4.50, 4.19, 4.01, 3.99, indicating the presence of four −OCH_3_ groups. ^1^H-^1^H COSY and NOE correlations were also in support of the structure of **1**. The COSY correlations between *δ* 7.71 (1H, *d*, *J* = 8.8 Hz, H-8) are interacting with *δ* 7.18 (1H, *dd*, *J* = 8.8 and 2.6 Hz, H-7) as well as long range with *δ* 8.72 (1H, *s*, H-5). In the difference NOE experiments, on irradiation of the proton at *δ* 8.72 (H-5), the signal intensities of both protons at *δ* 3.99 (H-6-OMe) (strong) and *δ* 4.19 (H-4-OMe) were enhanced significantly, indicating that two methoxy groups were located at C-4 and C-6. However, upon irradiation of *δ* 7.71 (H-8), the protons at *δ* 7.18 (H-7) and *δ* 7.16 (H-9) were enhanced. Furthermore, the NOE effect showed that the H-7 (*δ* 7.18) signal was enhanced upon irradiation of 6-OMe (*δ* 3.99), suggesting the placement of the aromatic methoxy group at C-6. The key HMBC correlations from aromatic proton H-5 (*δ* 7.18) to C-4a (*δ* 127.7), C-4b (*δ* 115.9), C-1 (*δ* 109.5), indicated the obviously chemical shift of the quaternary carbon, especially position 1 unambiguously [4–6]. Additionally, the HMBC correlations between the aromatic protons H-7 (*δ* 7.18), H-8 (*δ* 7.71) and H-9 (7.16) to (C-4a, C-6, C-8a) and (C-4a, C-6, C-7, C-8a, C-9) and (C-4a, C-4b, C-8, C-8a), respectively, established the phenanthrene of aristolactam-type alkaloid skeleton. In addition, the EIMS mass spectrum (found *m/z* 339, [M^+^]) showed typical aristolactam structure. The key fragmentation ions in the mass spectrum at *m/z* 324, 198, 171 and 170 were useful to obtain the structure of **1** (Figure 2). The principal ions were associated with loss of methyl and carbonyl derived from initial cleavages around the methoxy functions [9]. The presence of the methyl groups were confirmed by the fragment ions at *m/z* 324. In addition, the fragment ions at *m/z* 198 (M^+^-Me-Me-4CO) and *m/z* 171 (M^+^-Me-Me-4CO-HCN) indicated the presence of the amide group. Moreover, methylation of a known alkaloid **2** was performed to confirm that compound **1** is its methyl derivative. On the basis of the spectral data, the structure of compound **1** was recognized as 10-amino-2,3,4,6-tetramethoxyphenanthrene-1-carboxylic acid lactam (enterocarpam-III). This compound is being reported for the first time from a natural plant source.

**Figure 3:**
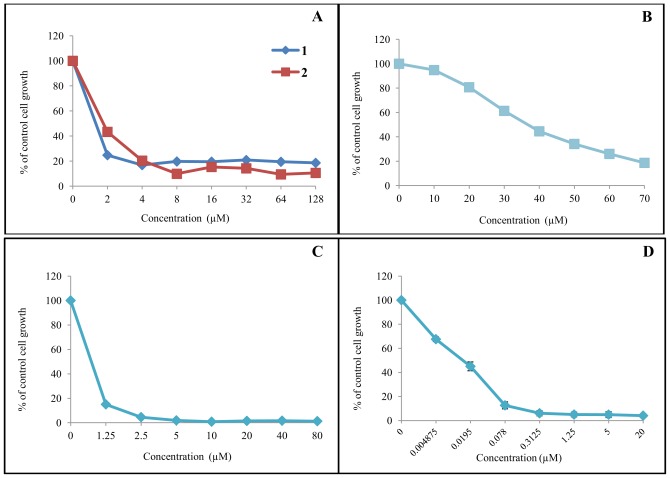
IC_50_ evaluations on HCT15 cells. Dose-response curves between the compound concentrations and percentages of control cell growth were plotted. IC_50_ values were derived using the software Curve Expert 1.4. The data represent the mean value ± standard deviation of three independent experiments performed in triplicate. (**A**) The IC_50_ values measured for compounds **1** and **2** were 1.68 ± 0.07 and 1.32 ± 0.03 μM, respectively. (**B**) The IC_50_ value of carboplatin was 37.2 ± 4.4 μM. (**C**) The IC_50_ value of gemcitabine was 0.74 ± 0.07 μM. (**D**) The IC_50_ value of vinorelbine was 0.018 ± 0.002 μM.

We investigated whether these compounds could have cytotoxic activities in human colon adenocarcinoma cell line HCT15 using the Sulforhodamine B (SRB) colorimetric assay. The 50% growth inhibitory concentrations (IC_50_) were determined from the dose-response relationships between the compound concentrations and the percentage of growth inhibitions (Figure 3). The IC_50_ values measured for compounds **1** and **2** were 1.68 ± 0.07 and 1.32 ± 0.03 μM, respectively. These data represent the mean value ± standard deviation of three independent experiments performed in triplicate. We also used the same system to test some established cancer therapeutic agents such as carboplatin, gemcitabine, and vinorelbine; the IC_50_ values measured for these compounds were 37.2 ± 4.4, 0.74 ± 0.07 and 0.018 ± 0.002 μM, respectively.

## 3. Experimental Section

### 3.1. General

Melting points were measured on a Büchi 322 micro melting point apparatus and were uncorrected. Infrared spectra were recorded as KBr pellets using a Shimadzu 8900 FTIR spectrophotometer. Ultraviolet absorption spectra were measured in methanol solution on Shimadzu 1601 spectrophotometer. High resolution mass spectra (electrospray ionization mode, ESI-MS) were measured on a micromass Q-TOF-2™ (Waters) spectrometer. Low resolution mass spectra were recorded on a Thermo Finnigan Polaris Q mass spectrometer at 70 eV (probe) for EIMS carried out with a Bruker Esquire. The ^1^H and ^13^C-NMR (1D and 2D) spectra were recorded using a Bruker DPX 400 spectrometer and were recorded as *δ* value in ppm down field from TMS. Silica gel 60 H (E. Merck.70–230 mesh ASTM, cat. No.7734.) was used for column chromatography (CC). Solvents for extraction, chromatography and recrystallization were distilled before uses. Fractions obtained from CC were mornitored by TLC (pre-coated silica gel 60 F_254_, 20 × 20 cm, MERCK).

### 3.2. Plant Material

The leaves and twigs of *O. enterocarpa* were collected from Prajeanburi, Province in Thailand, in March 2009 and were identified by N. Nuntasaen. A voucher specimen (BKF no.151499) was deposited at the Forest Herbarium, Department of National Park, Wildlife and Plant Conservation, Ministry of Natural Resources and Environment, Bangkok, Thailand.

### 3.3. Isolation and Extraction

Air-dried leaves and twigs of *O. enterocarpa* (2.7 kg) were ground and then defatted successively with hexane (3 × 19 L) and extracted subsequently with methanol-dichloromethane (3:1) (11 × 52 L.) Removal of the solvent from each extract under reduced pressure gave the crude hexane (15.83 g) and methanol-dichloromethane (211.18 g) extracts, respectively. The methanol-dichloromethane extract (211.2 g) was further separated by flash CC over silica gel (Merck 7737, Mesh 70–230, 700 g). Gradient elution was conducted initially with n-hexane, gradually enriched with ethyl acetate, followed by increasing amount of methanol in ethyl acetate and finally with methanol to afford eleven fractions, F_1_–F_11_. Fraction F_4_ (16.6 g) was rechromatograpged by CC over silica gel eluted with gradient system between hexane, ethyl acetate and methanol to give five subfractions, A_1_–A_5_. The pale yellow precipitate in subfraction A_4_ was recrystallized from ethanol to obtain compound **1** (0.28 g). Fraction F_4_ (3.8 g) further separated by CC over silica gel using gradient elution of hexane, ethyl acetate and methanol to obtain four subfractions, B_1_–B_4_. The subfraction B_3_ (0.38 g) then underwent repeated purified with the same procedure to afford four subfractions, C_1_–C_4_. The yellow precipitate in subfraction C_2_ was recrystallized from ethanol to give compound **2** (0.07 g).

### 3.4. Enterocarpam-III (**1**)

Yellow crystals, m.p. 183–184 °C; UV λ_max_ (MeOH) (log ɛ): 313(3.17), 359 (2.77), 386 (2.74) and 550 (1.74) nm; IR (KBr) ν (cm^−1^): 3464, 3167, 1699, 1682, 1654, 1614, 1568, 1533, 1483, 1475, 1439, 1201, 1157. ^1^H NMR (CDCl_3_, 400 MHz) and ^13^C NMR (CDCl_3_, 100 MHz): Table 1. HRESIMS *m/z* 340.1184 [M + H]^+^ (calcd for C_19_H_17_NO_5_ + H, 340.1185). EIMS *m/z* (rel.int.%): 339 (100), 324 (28), 310 (17), 296 (15), 281 (19), 266 (24), 210 (18), 198 (12), 171 (10) and 170 (9). COSY correlations H/H: 7/8; 8/7. HMBC correlations: see Figure 1.

### 3.5. Stigmalactam (**2**)

Yellow crystals, m.p. 275–276.5 °C [8], UV λ_max_ (MeOH) (log ɛ): 394(2.34), 298 (2.62) and 242 (2.90) nm; IR (KBr) ν (cm^−1^): 3550, 3431, 1679, 1614, 1599, 1419, 1332, 1236, 1195, 1061. ^1^H NMR (Acetone-*D*_6_, 400 MHz) and ^13^C NMR (Acetone-*D*_6_, 100 MHz): Table 1. HRESIMS *m/z* 326.1029 [M + H]^+^ (calcd for C_18_H_14_NO_5_ + H, 326.1028). EIMS *m/z* (rel.int.%): 325 (100), 310 (20), 307 (22), 292 (19), 282 (24), 279 (14), 267 (16), 264 (25), 239 (13). COSY correlations H/H: 5/7; 7/5, 7/8; 8/7. HMBC correlations: see Figure 1.

Methylation of Stigmalactam: To a solution of stigmalactam (3.9 mg, 0.012 mmol) in anhydrous acetone (5 mL) was added excess MeI and the mixture was stirred at room temperature for 4 h. The crude reaction mixture was filtered and concentrated *in vacuo*. The crude product was purified by preparative thin layer chromatography (EtOAc-hexane = 4:6 as developing solvent) gave enterocarpam-III (**1**) (3.7 mg, 91% yield).

### 3.6. Cytotoxicities

#### 3.6.1. Cell Lines and Culture Conditions

The human colon adenocarcinoma cell line HCT15 was obtained from American Type Culture Collection (Rockville, MD). The HCT15 cells were grown in Roswell Park Memorial Institute medium 1640 (RPMI 1640) with 10% fetal bovine serum (FBS) and 1% antibiotics (50 units/mL penicillin, 50 μg/mL streptomycin). The HCT15 cells were grown as monolayer at 37 °C in a humidified atmosphere of 5% CO_2_ and 95% air.

#### 3.6.2. Sulforhodamine B (SRB) Colorimetric Assay

Cell survival was determined by using the colorimetric Sulforhodamine B assay. The assay relies on the ability of Sulforhodamine B to bind to protein components of cells that have been fixed to tissue-culture plates by trichloroacetic acid (TCA). Sulforhodamine B is bright-pink aminoxanthane dye with two sulfonic groups that bind to basic amino-acid residues under mild acidic conditions, and dissociate under basic conditions. As the binding of Sulforhodamine B is stoichimetric, the amount of dye extracted from stained cells is directly proportional to the cell mass.

Sulforhodamine B Assay Protocol: The growth inhibition of HCT15 cells was determined using the Sulforhodamine B (SRB) assay according to a published protocol. Briefly, after adjusting the cell concentration with Roswell Park Memorial Institute medium 1640 (RPMI 1640) supplemented with 10% fetal bovine serum (FBS) and 1% antibiotics (50 units/mL penicillin, 50 μg/mL streptomycin) to obtain seeding density at 1 × 10^4^ cells/well, the cell suspensions were seeded in 96-well tissue-culture plates, which were pre-added with various concentrations of the test sample. The cell suspensions were occasionally mixed during plating to ensure an even distribution of the cells. The cells were allowed to attach for 2–3 h at 37 °C in a humidified incubator with 5% CO_2_. The first plate, as a no-growth control (day 0), was proceeded to fix the cell monolayer, whereas the remaining plates were incubated at 37 °C in a humidified incubator with 5% CO_2_ for 72 h. At the end of each treatment, the cells were fixed by gentle addition of 100 μL of cold 10% (wt/v) trichloroacetic acid (TCA) to each well, followed by incubation at 4 °C for 1 h. Plates were washed four times with deionized water and removed excess water using paper towels. The plates were then left to air dry at room temperature (20–25 °C). Cells were stained with 100 μL of SRB solution (0.057% SRB wt/v in 1% acetic acid), which was added to each well for 30 min at room temperature. The plates were then quickly rinsed four times with 1% (v/v) acetic acid to remove unbound dye and allowed to dry at room temperature. Next, bound dye was solubilized with 10 mM Tris base solution (pH 10.5). The optical density (OD) was read at 510 nm using a fluorescence plate reader (Biotech K40). The absorbance values of treated samples were calculated as the percentage of cell-growth inhibition using the formula below:

$$\begin{array}{c} \%\text{of control cell growth}=\frac{\text{mean OD sample}-\text{mean}\;\text{OD day }0\times 100}{\text{mean OD}\;\text{neg}.\;\text{control}-\text{mean OD day }0}\\ \%\text{growth inhibition}=100-\%\text{of control cell growth} \end{array}$$

For IC_50_ determination, a dose-response curve between the compound concentration and percent of control cell growth was plotted. IC_50_ values were derived using the software Curve Expert 1.4.

## 4. Conclusions

The present study demonstrated, for the first time, that enterocarpam-III and stigmalactam isolated from the leaves and twigs of *O. enterocarpa*, possesses in cytotoxicities. As seen from the IC_50_ values, our compounds were within the range of these established cancer therapeutic agents. Therefore, these compounds could potentially be useful as chemotherapeutic agents, but much further investigation is still needed.

## Figures and Tables

**Table 1:** NMR chemical shifts (*δ*) of compounds **1** and **2** (^1^H: 400 MHz and ^13^C: 100 MHz).

Position	1 (CDCl_3_)		2 (acetone-*D*_6_)	

	*δ*_H_ m (*J* = Hz)	*δ*_C_ (DEPT)	*δ*_H_ m (*J* = Hz)	*δ*_C_ (DEPT)
1	-	109.5 (C)	-	109.8 (C)
2	-	154.2 (C)	-	149.2 (C)
3	-	146.0 (C)	-	144.0 (C)
4	-	157.1 (C)		158.3 (C)
4a	-	127.7 (C)	-	129.1 (C)
4b	-	115.9 (C)	-	123.5 (C)
5	8.72 *s*	108.5 (CH)	8.75 *d* (2.7)	109.2 (CH)
6	-	157.6 (C)	-	150.3 (C)
7	7.18 *dd* (8.8, 2.6)	116.1 (CH)	7.20 *dd* (8.8, 2.7)	116.8 (CH)
8	7.71 *d* (8.8)	129.7 (CH)	7.80 *d* (8.8)	130.5 (CH)
8a	-	127.8 (C)	-	129.1 (C)
9	7.16 *s*	106.4 (CH)	7.15 *s*	104.8 (CH)
10	-	131.8 (C)	-	133.9 (C)
10a	-	126.1 (C)	-	128.5 (C)
11	9.30 *brs*	-	9.20 *s*	-
12	-	167.7 (C)	-	167.3 (C)
2-OMe	4.50 *s*	63.1 (CH_3_)	4.55 *s*	63.0 (CH_3_)
3-OMe	4.01 *s*	61.7 (CH_3_)	4.00 *s*	55.7 (CH_3_)
4-OMe	4.19	60.8 (CH_3_)	4.20 *s*	60.2 (CH_3_)
6-OMe	3.99 *s*	55.4 (CH_3_)	-	-
6-OH	-	-	8.35 *s*	-
